# Role of Vitamin D in reducing number of acute exacerbations in Chronic Obstructive Pulmonary Disease (COPD) patients

**DOI:** 10.12669/pjms.333.12397

**Published:** 2017

**Authors:** Dur Muhammad Khan, Aziz Ullah, Fawad Ahmad Randhawa, Somia Iqtadar, Nasir Farooq Butt, Khadija Waheed

**Affiliations:** 1 Dr. Dur Muhammad Khan, FRCP. Department of Medicine, King Edward Medical University, Lahore, Pakistan; 2 Dr. Aziz Ullah, Department of Medicine, King Edward Medical University, Lahore, Pakistan; 3 Dr. Fawad Ahmad Randhawa, FCPS, MCPS, FCPS. Department of Medicine, King Edward Medical University, Lahore, Pakistan; 4 Dr. Somia Iqtadar, FCPS. Department of Medicine, King Edward Medical University, Lahore, Pakistan; 5 Dr. Nasir Farooq Butt, MCPS, FCPS. Department of Medicine, King Edward Medical University, Lahore, Pakistan; 6 Dr. Khadija Waheed, FCPS. Department of Gynecology & Obstetrics, Department of Gynecology & Obstetrics King Edward Medical University, Lahore, Pakistan

**Keywords:** Exacerbation, Vitamin D, COPD, FEV1, FVC

## Abstract

**Background and Objective:**

Chronic obstructive pulmonary disease (COPD) is characterized by chronic incompletely reversible poor airflow and air trapping and usually this debilitating disorder limits the outside activities of the patients depriving them of sunlight which is a rich source of Vitamin D. The objective of this study was to determine the effect of vitamin D supplementation in reducing number of acute exacerbation in COPD patients.

**Methods:**

This randomized control trial was conducted at East Medical Ward Mayo Hospital Lahore from January to December 2015 as exacerbations of COPD are season dependent. Diagnosis was confirmed by performing Pulmonary Function Tests (PFTs). Basic demographical information was obtained and baseline PFTs of the patient was done. Only Group A patients was treated with oral vitamin D intake of 2000 IU daily for 6 months. Vitamin D level was measured at 0, 2, 4, and 6 months and exacerbation of COPD, FEV1 and FVC was measured weekly. Both the groups were given standard treatment for exacerbation of COPD. Spirometry was repeated at each visit. Blood samples were collected every 2 months for vitamin D. Supplementation was stopped if vitamin D level exceeded 100ng/ml.

**Results:**

The mean age of the patients was 46.28±8.83 years, the male to female ratio was 1.8:1. The mean 25(OH) level at baseline was 24.08±2.58 and at 6th month was 29.60±8.74. The mean FVC at baseline was 77.83±5.49 and at 6th month was 91.34±5.52. The exacerbation at baseline was present in all 120(100%) patients and at 6th month was reduced to 4(3.3%).

**Conclusion:**

Vitamin D supplementation has significant effect in reducing number of acute exacerbation in COPD patients when it is given for prolonged period.

## INTRODUCTION

The main reason for Vitamin D deficiency is lack of ultraviolet-B radiation from less sun contact, and this result in lack of vitamin D production in skin. About 3% of the human genome regulates by vitamin D endocrine system.^1^ Vitamin D is currently of great public health interest, because its deficiency is common and is causally associated with musculoskeletal diseases. Half of the world’s population is affected by vitamin D insufficiency.^2^,^3^

Hypovitaminosis D is related with COPD according to Quint JK and in the general population increased susceptibility to infection.^4^ Emerging evidence indicates that vitamin D-mediated innate immunity, chiefly by improved expression of the human antimicrobial peptide, is significant in defense from against respiratory tract pathogens. Control of respiratory tract infection through correction of vitamin D level may result in better outcome in patients with Chronic obstructive pulmonary disease (COPD).^5^

Few studies have measured the significance of vitamin D deficiency in COPD by calculating serum levels of 25-hydroxyvitamin D (25-[OH]D), which is the important circulating vitamin D metabolite and recognized as the finest short-term biomarker of entire contact to vitamin D. With disease development, marked by decay in FEV1, patients grow systemic significances and became prone to infectious exacerbations which are precipitated by concomitants vitamin D deficiency.^6^

Deficiency in 25-hydroxyvitamin D is related with emerging risk of infections containing influenza, TB and pneumonia.^7^ COPD is categorized by permanent expiratory airflow restriction. The disease is intermingled with times of exacerbations that have necessary results for patients and health care providers. Mainly Exacerbations are triggered due to infection.^8^

Of note, low serum 25-hydroxyvitamin D (25-[OH]D) levels, reproducing vitamin D status, are related with reduced FEV1, and it is present in 60% to 75% of severe COPD patients.^2^

According to the Vitamin D Council, vitamin D levels above 30 - 40 ng/mL may reduce the risk of COPD. To reach these levels, most people need to take 1000–5000 international units (25–125 mcg) per day of vitamin D3, an active form of vitamin D that is produced under the skin. However, they also stress that, because there is considerable variation from person to person, proper dosage should be determined by measuring a patient’s vitamin D blood levels before, and several months after, taking vitamin D3 additions or increasing ultraviolet-B exposure.^9^

An attractive target for intervention studies are particularly for COPD, the vitamin D pathway is due to lack of vitamin that can increase chronic airway and systemic inflammation, decrease bacterial clearance, and urge the chance for infectious exacerbations at the same time.^10^ Our aim was to explore the effect of vitamin D supplementation on COPD exacerbations.

## METHODS

Data was collected after approval of synopsis from ethical committee. After taking informed consent, all patients were enrolled in this study from medical units of Mayo Hospital Lahore. Basic demographical information (age, gender, occupation, etc.) was obtained and baseline pulmonary function test (PFTs) of the patient was done.

### Inclusion criteria

Patients having age between 18-60Y of either gender, Patients with normal BMI i.e. BMI = 19-24.5 kgm^-2^, FEV_1_ less than 80% of predicted & forced expiratory volume in first second FEV1/ forced vital capacity (FVC) < .7 and patients with vitamin D levels below 30 ng/ml.

### Exclusion criteria

Patients with a history of asthma, bronchiectasis, carcinoma of the bronchus, or other significant respiratory disease. Patients having Serum calcium >10.5 mg/dL. Those with history of diseases like nephrolithiasis, hypercalciuria, malignancy, tuberculosis, sarcoidosis, Paget’s disease, malabsorption syndromes. Hepatic or renal insufficiency (creatinine >1.5 mg/dl and estimated creatinine clearance <20 ml/minute), Use of active metabolites of vitamin D within 6 months of screening and female patients on contraceptives.

Patients were divided randomly in two groups A and B. Group A patients received oral vitamin D 2000 IU daily for 6 months. Vitamin D level was measured at 0, 2, 4, and 6 months and exacerbation of COPD, FEV1 and FVC was measured weekly. Acute exacerbation of COPD was defined as “A decline in the base line FEV1 by 10 % or more for a particular patient

Time to first exacerbation was assessed by quantifying the days between randomization and the first exacerbation. Both the groups were given standard treatment for exacerbation of COPD. To obtain data on exacerbations, researchers asked the participants to complete diaries weekly with FEV1, FVC, detailed respiratory tract symptoms, visits to health care providers, hospitalizations, and changes in medication. At each visit, diaries were reviewed in the participant’s presence. Spirometry was repeated at each visit by using standard equipment. Blood samples were collected every 2 months to measure serum vitamin D levels. Vitamin D supplementation was stopped in patient whose serum vitamin D level exceeded 100ng/ml (optimum level). All the data was collected by the researcher himself on a prescribed proforma.

All data was entered and analyzed using SPSS version 20. Qualitative data was presented in form of frequency (%). Quantitative data was presented in form of Mean ± S.D. P-value < 0.05 was considered as significant.

## RESULTS

In this study total 120 cases were enrolled. The mean age of the patients was 46.28±8.83 years with minimum and maximum ages of 30 & 60 years respectively. In our study 78(65%) patients were male and 42(35%) patients were females. The male to female ratio of the patients was 1.8:1. In our study the 97(80.8%) patients were smokers and 23(19.2%) patients were non-smoker and the mean BMI of the patients was 22.57±1.72 kg/m2. The study results showed the mean 25(OH) level which is depicted in Table-I. The mean FEV1 at baseline was 67.54±5.50, at 2nd month was 71.07±5.68, at 4th month was 74.18±6.81 and at 6th month was 78.97±6.94. There was significant increase in FEV1 of patients after 6 months. The study results showed that the mean value of FVC at baseline was 77.83±5.49, its mean value at 2nd month was 89.75±7.77, at 4th month its mean value was 89.75±7.77 and its mean value at 6th month was 91.34±5.52. The difference was significant. In our study the dyspnea at baseline was present in 96(80%) patients, at 2nd month it was present in 96(80%) patients, at 4th month it was present in 91(75.8%) patients and at 6th month it was present in 87(72.5%) patients. In our study the sputum volume at baseline was normal in 47(39.2%) patients, at 2nd month it was normal in 47(39.2%) patients, at 4th month it was normal in 93(77.5%) patients and at 6th month it was normal in 109(90.8%) patients. In this study the sputum purulence at baseline was present in 96(80.0%) patients, at 2nd month it was present in 96(80%) patients, at 4th month it was present in 90(75.0%) patients and at 6th month it was present in 79(65.8%) patients. The cough at baseline was present in all (100%) patients, at 2nd month it was present in 118(98.3%) patients, at 4th month it was present in 114(95%) patients and at 6th month it was present in 103(85.8%) patients. In this study the wheezing at baseline was present in all patients, at 2nd month it was present in 95(79.2%) patients, at 4th month it was present in 76(63.3%) patients and at 6th month it was present in 14(11.7%) patients. The study results showed that the exacerbation at baseline was present in all 120(100%) patients, at 2nd month it was present in 79(65.8%) patients, at 4th month it was present in 47(39.2%) patients and at 6th month it was present in 4(3.3%) patients. Statistically, insignificant difference was found between the study group with dyspnea. Statistically insignificant difference was found between the study group with sputum purulence i.e. p-value>0.05. The exacerbation was present in all cases as is shown in Table-II. Statistically insignificant difference was found between the study groups in earlier follow-ups but it was significant at final follow-up.

**Table-I:** Descriptive statistics of 25(OH) level from baseline to 6th month.

		*At baseline*	*2nd month*	*4th month*	*6th month*
25(OH) level	N	120	120	120	120
	Mean	24.08	26.37	31.15	29.60
	SD	2.58	2.63	5.22	8.74
	Minimum	20.00	22.00	22.30	16.00
	Maximum	29.00	31.00	40.00	46.00

**Table-II:** Comparison of exacerbation from baseline to 6th month with study groups.

			*Study Groups*		*Total*	*p-value*

			*A*	*B*		
Exacerbation	Baseline	Present	60	60	120	0.999
		Absent	0	0	0	
	2nd month	Present	39	40	79	0.847
		Absent	21	20	41	
	4th month	Present	24	23	47	0.852
		Absent	36	37	73	
	6th month	Present	0	4	4	0.042
		Absent	60	56	116	

## DISCUSSION

The study determined the effect of vitamin D supplementation in reducing number of acute exacerbation in” COPD” patients. Vitamin D deficiency is prevalent amongst patients with chronic obstructive pulmonary disease (COPD) and comes to be more frequent with increased disease severity. Chronic obstructive pulmonary disease (COPD) is a common preventable and treatable disease, characterized by persistent air flow limitation that is usually progressive and associated with an enhanced chronic inflammatory response of airways and the lungs to noxious particles or gases; exacerbation and comorbidities contribute to the overall severity in individual patients.^11^

In our study the mean 25(OH) level at baseline was 24.08±2.58 and at 6th month was 29.60±8.74. The mean FEV1 at baseline was 67.54±5.50 and at 6th month was 78.97±6.94. The mean FVC at baseline was 77.83±5.49 and at 6th month was 91.34±5.52. The exacerbation at baseline was present in all 120(100%) patients and at 6th month it was present in 4(3.3%) patients. Statistically insignificant difference was found between the dyspnea, sputum volume, sputum purulence, cough, fever, common cold, wheezing, sore throat but for exacerbation, the difference was significant. Quint JK reported that 25-hydroxyvitamin D deficiency is associated with COPD and increased susceptibility to infection in the general population.^4^ Lehouck et al.found that the median time to first exacerbation did not differ significantly between the studied groups, nor did exacerbation rates, FEV1, hospitalization, quality of life, and death.^12^ However, a post hoc analysis in 30 participants with severe vitamin D deficiency (serum 25-(OH) D level <10 ng/ml at baseline) showed a significant reduction of exacerbations in the vitamin D group. A recent randomized trial looking at the effect of vitamin D supplementation in patients with COPD did not show a statistically significant reduction in exacerbation rates. A subgroup of patients with very low vitamin D levels did show some reduction in COPD exacerbation rates. In participants with severe vitamin D deficiency at baseline, supplementation may reduce exacerbations.^12^

According to a recent meta-analysis, the benefits of supplementation were only present when baseline 25-OHD levels are very low (<10 ng/ml).^13^ One more study compared the effects of vitamin D and placebo on FEV1 and exacerbation rate in the patients with moderate to very severe COPD. They reported that this dose of vitamin D had not improved FEV1 and exacerbation rate. This difference may be due to the difference between baseline serum vitamin D levels in these studies.^14^ For COPD, the vitamin D pathway is an attractive target for intervention studies because vitamin D deficiency may enhance chronic airway and systemic inflammation, reduce bacterial clearance, and increase the risk for infectious exacerbations at the same time.^10^ Deficiency in 25-hydroxyvitamin D results from a number of causes and is associated with increased risk of infections including influenza, TB and pneumonia.^7^ On contrary Rezk et al.^15^ in their study found a significant improvement in dyspnea scale (p < 0.003), coupled with a decrease in disease exacerbations (p < 0.001) and CRP (p < 0.001) a year after vitamin D replacement. However, the FEV1 and FVC did not differ significantly. One more study by Abolfazl Zendedel et al.^16^ have reported that after the intervention, there were significant differences in FEV1 and the number of COPD exacerbation between the case and control group patients. Also, after the study, in the case group, FEV1 was increased and the number of COPD exacerbation was decreased significantly.

## CONCLUSION

Vitamin D can be beneficial in reducing exacerbations but it exerts this effect when it is taken in adequate amount and moreover for a prolonged period of time. Moreover, not every symptom benefitted with vitamin D replacement. So, further studies should be encouraged with different methodology and design to prove benefits of vitamin D in COPD.

### Authors’ Contribution

**DMK** conceived and designed the manuscript.

**AU and NFB** did data collection and manuscript writing.

**FAR and SI** did statistical analysis & editing of manuscript.

**KW** did review and final approval of manuscript.
